# An agricultural community’s perspectives on COVID-19 testing to support safe school reopening

**DOI:** 10.3389/fpubh.2023.1215385

**Published:** 2023-08-03

**Authors:** Magaly Ramirez, Sonia Bishop, Genoveva Ibarra, Parth Shah, Miriana C. Duran, Hwa Young Chae, Laurie Hassell, Lorenzo Garza, Sandra Linde, Michelle M. Garrison, Paul K. Drain, Linda K. Ko

**Affiliations:** ^1^Department of Health Systems and Population Health, University of Washington School of Public Health, Seattle, WA, United States; ^2^Center for Community Health Promotion, Fred Hutchinson Cancer Center, Sunnyside, WA, United States; ^3^Fred Hutchinson Cancer Center, Seattle, WA, United States; ^4^Institute of Translational Health Sciences, Seattle, WA, United States; ^5^Sunnyside School District, Sunnyside, WA, United States; ^6^Astria Health, Sunnyside, WA, United States; ^7^Department of Public Health, Purdue University College of Health and Human Sciences, West Lafayette, IN, United States; ^8^Department of Global Health, University of Washington School of Public Health, Seattle, WA, United States; ^9^Department of Medicine, University of Washington School of Medicine, Seattle, WA, United States

**Keywords:** agricultural community, rural community, community perspectives, COVID-19 testing, COVID-19 transmission, school reopening, in-person learning, COVID-19 mitigation

## Abstract

**Introduction:**

School-based COVID-19 testing may be an effective strategy for reducing transmission in schools and keeping schools open. The study objective was to examine community perspectives on school-based COVID-19 testing as a mitigation strategy to support safe school reopening.

**Methods:**

We conducted a qualitative study in Yakima County, an agricultural region of Washington state, where over half of residents are Hispanic/Latino. From June to July 2021, we interviewed 18 students (13 years old, on average) and 19 school employees, and conducted four focus groups (2 in Spanish, 2 in English) with 26 parents. We audio-recorded the semi-structured interviews and focus group discussions which were then transcribed. We used an inductive, constant comparison approach to code the transcripts and conducted a thematic analysis to generate themes.

**Results:**

We identified four main themes. Students, parents, and school employees desired a return to in-person learning (Theme 1). Schools implemented numerous COVID-19 mitigation strategies (e.g., masking) to facilitate a safe return to school but felt that adding testing would not be feasible due to a lack of resources and overworked staff (Theme 2). Parents and school employees’ familiarity with COVID-19 testing procedures influenced their support for testing (Theme 3). Parents and school employees felt there were inadequate resources for individuals who test positive for COVID-19 (Theme 4).

**Discussion:**

Schools require adequate resources and medical personnel to implement COVID-19 testing. Individuals also need resources after testing positive, including physical space to isolate, financial resources for those without paid time off, and delivery of food and other necessities to households in rural communities.

## 1. Introduction

In March 2020, all U.S. states enacted school closures to mitigate Coronavirus Disease 2019 (COVID-19) transmission (1). Beyond providing academic instruction, schools are an essential resource for children’s physical, mental, and social health needs. With schools closed, social inequities that existed prior to the COVID-19 pandemic were exacerbated (2). Consequently, the American Academy of Pediatrics urged a safe return to in-person learning (3). In response, state officials developed policies based on the Centers for Disease Control and Prevention guidelines to reopen schools safely by implementing COVID-19 mitigation strategies.

COVID-19 mitigation strategies (e.g., masking, social distancing) were widely implemented in schools to support a safe return to in-person learning, but acceptance of these strategies among under-resourced communities and communities of color was not fully explored. During the COVID-19 pandemic, public health communication errors (e.g., providing conflicting information) and disinformation campaigns undermined public trust in scientific information and increased public fear and confusion, especially among communities of color (4, 5). Therefore, understanding community perspectives on COVID-19 mitigation strategies, including fears, concerns, and/or questions, is critical to inform approaches that encourage community acceptance of COVID-19 mitigation strategies (6).

Rapid Acceleration of Diagnostics Underserved Populations (RADx-UP), an initiative by the National Institutes of Health, aims to accelerate implementation of COVID-19 testing in communities most affected by the pandemic (7). RADx-UP funded ReOpening Schools Safely and Educating Youth (ROSSEY), a community–academic partnership aiming to develop, test, and evaluate multi-level COVID-19 risk communication strategies. The strategies promote participation in school-based COVID-19 testing to enable students’ safe return to school and help schools stay open for onsite learning in Yakima County, Washington (8–10).

Yakima County is a rural agricultural community with a large population of migrants and farmworkers (11). Over 50% of Yakima County residents are Hispanic/Latino, 15% of residents live in poverty, and 28% of jobs are in agriculture (12, 13). In June 2020, Yakima County had the most COVID-19 cases *per capita* among West Coast counties. The outbreak was attributed to the work and housing conditions of agricultural workers (14).

To inform the development of community-appropriate COVID-19 risk communication strategies for ROSSEY, we conducted this qualitative study to identify the social, ethical, and behavioral needs of communities in Yakima County to safely return to school and maintain onsite learning. This paper describes the community’s perspectives on school-based COVID-19 testing as a mitigation strategy to support safe school reopening.

## 2. Materials and methods

We conducted semi-structured interviews with school employees (e.g., teachers, nurses, principals) and students as well as focus groups with parents in Yakima County. We engaged community representatives throughout the study as co-investigators, advisors, field managers, and community health workers (CHWs).

### 2.1. Ethical approval

The University of Washington Institutional Review Board approved the study (STUDY00013064). Study participants provided verbal or electronic informed consent. We obtained parental consent and child assent for student participants.

### 2.2. Participant recruitment

**Figure 1:**
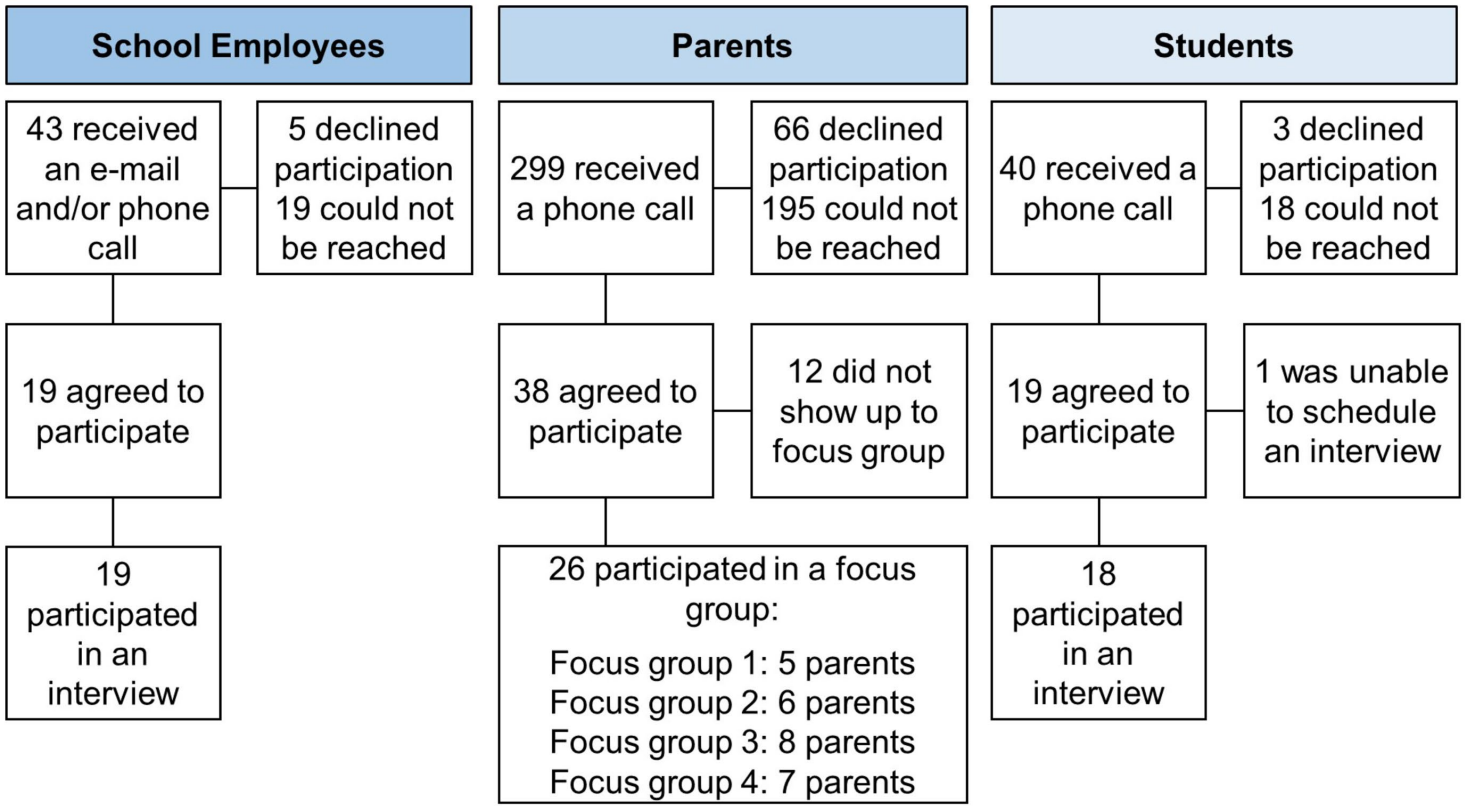
Diagram of participant recruitment, enrollment, and data collection.

Figure 1 illustrates participant recruitment. We used purposive sampling to recruit school employees. The research team generated a list of administrators, teachers, and support staff from school districts throughout Yakima County. Trained CHWs and research staff recruited participants via email and/or telephone and obtained consent for participation. CHWs were bilingual (English and Spanish) and bicultural.

We also used purposive sampling to recruit parents for the focus groups. We sent recruitment letters to a sample of parents who participated in the Together We STRIDE study (Strategizing Together Relevant Interventions for Diet and Exercise) and agreed to be contacted for participation in future studies; eligible parents had at least one child in grade K–8 (15). A week after the recruitment letters were sent, the CHWs contacted the parents to screen and obtain consent for participation. When 10 parents were enrolled, the CHWs scheduled a date and time for the focus groups. We convened four focus groups (2 in English and 2 in Spanish) with a total of 26 parents.

We asked parents who participated in a focus group whether they were interested in their child being interviewed by a member of the research team. If a parent agreed, a research team member contacted the parent to obtain parental consent and child assent and schedule an interview.

School employees and parents received a $30 gift card for compensation and students received a $15 gift card.

### 2.3. Setting and participant characteristics

We conducted school employee interviews via a Health Insurance Portability and Accountability Act (HIPAA)-approved virtual platform or phone call, parent focus groups via a HIPAA-approved virtual platform, and student interviews via a phone call. All interviewers and focus group moderators were trained and experienced in qualitative data collection. A CHW moderated the focus groups. Interviews and focus groups took place from June to July 2021. Table 1 provides characteristics of study participants. All students, 84% of parents, and 60% of school employees identified as Hispanic/Latino.

**Table 1:** Study participant characteristics.

Characteristic	School employees (*N* = 19)	Parents (*N* = 26)	Students (*N* = 18)
Age (years), mean (SD)		45.7 (6.9)	13 (1.0)
*Gender*^a^			
Male	5 (26.3%)	1 (3.8%)	8 (44.4%)
Female	12 (63.2%)	25 (96.2%)	10 (55.6%)
*Ethnicity*			
Hispanic/Latino	11 (57.9%)	22 (85.6%)	18 (100%)
Non-Hispanic/Latino	8 (42.1%)	4 (15.4%)	0 (0%)
*Interview language*			
Bilingual (Spanish dominant)	2 (10.5%)	3 (11.5%)	2 (11.1%)
Bilingual (English dominant)	8 (42.1%)	7 (26.9%)	11 (61.1%)
English only	9 (47.4%)	5 (19.2%)	5 (27.8%)
Spanish only	0 (0%)	11 (42.3%)	0 (0%)
*Annual household income*			
Less than $15,000	0 (0%)	2 (7.7%)	
$15,000–$34,999	0 (0%)	10 (38.5%)	
$35,000–$49,999	2 (10.5%)	6 (23.1%)	
$50,000–$74,999	2 (10.5%)	3 (11.5%)	
$75,000 or more	10 (52.6%)	2 (7.7%)	
Do not know	5 (26.3%)	3 (11.5%)	
*Country of origin*^a^			
United States	15 (78.9%)	10 (38.5%)	
Outside of the United States	3 (15.8%)	16 (61.5%)	
*Health insurance status*^a^			
Employer sponsored insurance	19 (100%)	7 (26.9%)	
Individual health insurance	0 (0%)	2 (7.7%)	
Medicare	0 (0%)	1 (3.8%)	
Medicaid, Washington Apple Health, or coupons	0 (0%)	4 (15.4%)	
Uninsured	0 (0%)	12 (46.2%)	
*Employment status*^a^			
Full time	19 (100%)	13 (50.0%)	
Part time	0 (0%)	7 (26.9%)	
Seasonal work	0 (0%)	1 (3.8%)	
Unemployed	0 (0%)	4 (15.4%)	
*Marital status*^a^			
Married/marriage-like relationship	15 (78.9%)	17 (65.4%)	
Single/divorced	3 (15.8%)	9 (34.6%)	
*Highest level of education*			
Elementary school	0 (0%)	5 (19.2%)	
Some high school	0 (0%)	6 (31.6%)	
High school graduate or GED	3 (15.8%)	4 (15.4%)	
Some college	4 (21.1%)	6 (23.1%)	
College graduate	4 (21.1%)	3 (11.5%)	
Graduate school degree	8 (42.1%)	2 (7.7%)	
*Common place to receive healthcare*^a^			
Doctor’s/nurse office	17 (89.5%)	6 (23.1%)	
Hospital	0 (0%)	1 (3.8%)	
Clinic	1 (5.3%)	18 (69.2%)	
Traditional medicine	0 (0%)	1 (3.8%)	
People per household, mean (SD)	3.2 (1.3)	5.5 (2.4)	

a Missing value because participant(s) preferred not to provide an answer.

### 2.4. Data collection

**Figure 2:**
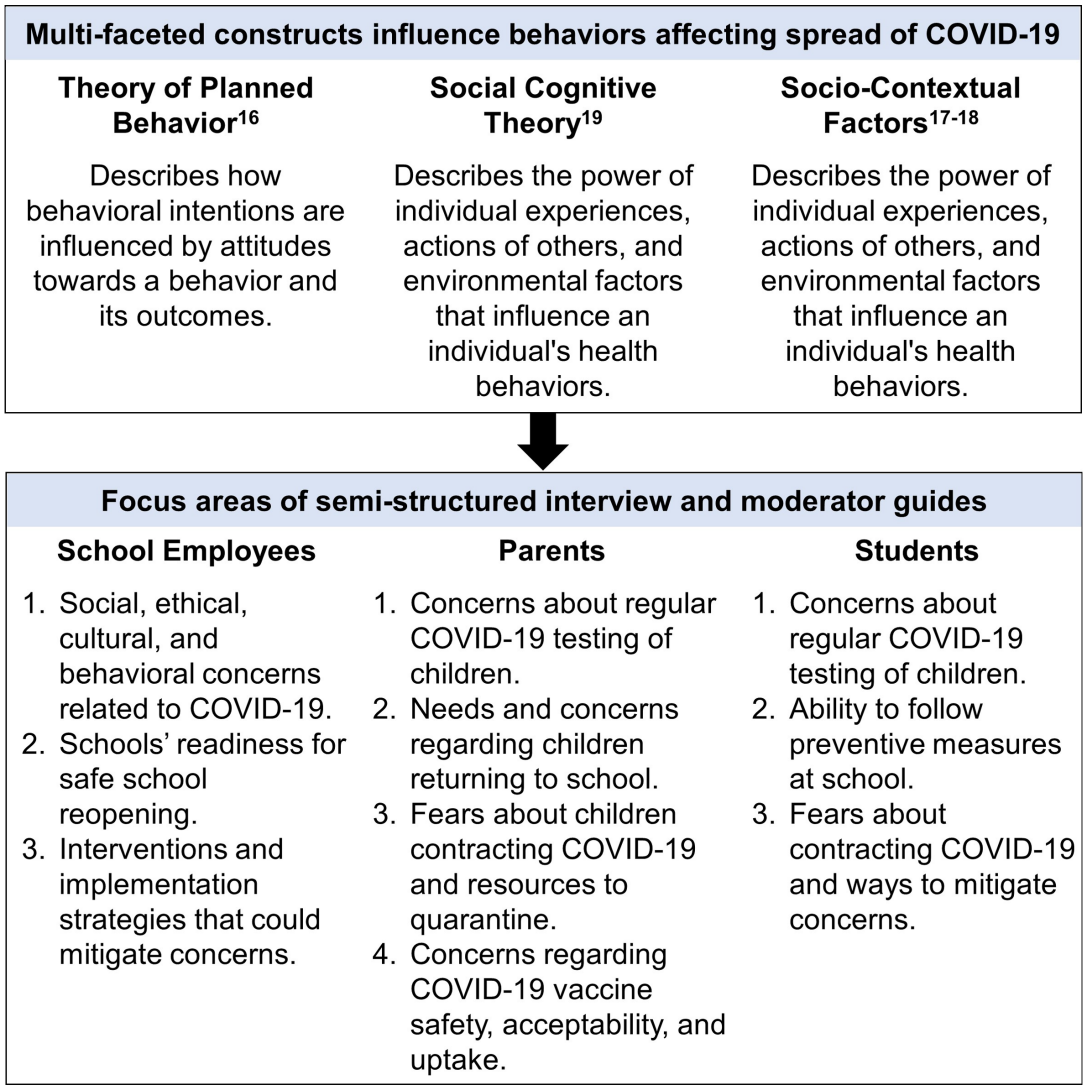
Focus areas of the interview and focus group moderator guides were informed by multiple theoretical frameworks (16–19).

Figure 2 shows the focus areas of the semi-structured interview and focus group guides, which were informed by the Theory of Planned Behavior, Social Cognitive Theory, and socio-contextual factors (16–19). A community advisory board provided feedback during development of the guides. A professional translated the focus group guide from English to Spanish and reviewed by English–Spanish bilingual staff members for accuracy. Interviews with school employees lasted 45–60 min, focus groups with parents lasted 60–90 min, and interviews with students lasted 25–30 min. We audio-recorded all interviews and focus groups which were transcribed and translated by a professional service.

### 2.5. Data analysis

We coded transcripts for school employees, parents, and children separately using Dedoose version 9.0.62 (Los Angeles, CA). We used an inductive, constant comparison approach to code the transcripts (20). Five members of the research team coded the transcripts using inductive coding, followed by deductive coding. We used *a priori* codes based on the interview and focus group guides to ensure that information from the questions was retained during coding. The team met weekly to refine the codebooks by adding, removing, and revising codes to address inter-rater agreements and compare new and existing data.

For the present study, we analyzed the set of codes regarding school-based COVID-19 testing. The first author (MR) identified themes from the codes by first reviewing the excerpts within each code and identifying tentative themes based on the content of the excerpts (21). The interrelationship across and within themes was analyzed, resulting in a collection of candidate themes. Next, MR reviewed the candidate themes with a research team member (MD). They refined the themes to ensure that excerpts within themes cohered and that each final theme was distinct from the others.

## 3. Results

We identified four main themes and 11 subthemes, which are shown in Table 2 with illustrative quotes. Parents, students, and school employees wanted students to return to in-person learning (Theme 1). School employees noted a lack of resources and overworked staff as barriers to adding COVID-19 testing to schools’ mitigation strategies (Theme 2). Parents and school employees’ familiarity with COVID-19 testing procedures influenced their support for testing (Theme 3). Parents and school employees felt there were inadequate resources for individuals who test positive for COVID-19 (Theme 4).

**Table 2:** Illustrative quotes from study participants, by theme and subtheme.

Theme 1: there is a shared desire for students to return to in-person learning	
Subtheme 1a: home environment made it difficult for students to focus on remote learning	It’s true, the children’s grades, at least with mine, they went down. Definitely much lower than the grades they would always have, because exactly, they were always distracted with something else. They were always getting snacks, going to the bathroom. Their minds were not focused on the computer, their minds were focused on something else, other temptations to go do, to go learn something else, the cat, the dog, or whatever it was, but there were always distractions. They were not focused on their—they focused about 30%, but everything else was distraction. *Parent* I do not know, there was just a lot of things to distract me and stuff … like my parents being home. Them telling me to do stuff. And then also the internet sometimes shutting off and stuff. *Student* The distractions. Because when we are at home it’s the TV, video games, that kind of stuff. *Student* There was parents who were expecting their children to do chores for them while they were supposed to be in class. Um, like things they would never do in the school year. They would never come to my classroom and say, “Hey, I need you to unload the dishwasher,” but they would do that when we were on Zoom. *School Employee* I mean, you have got … families of six or seven kids, and they are at home and you live in a small, little house, multi-generational, the kid does not even have a place to sit down to be quiet and have a quiet place or a place for them to keep their stuff. And, you know, you have got kids yelling at each other—siblings, they are siblings fighting over it and yeah, you know? *School Employee*
Subtheme 1b: quality of remote teaching was lower compared to in-person teaching	I think one of the things that was hardest, at the beginning it seemed novel and interesting, but as time went on teachers did their best, but teachers I do not think really were trained how to teach online. So they were trying to do the same thing that they would do in person and they were losing the interest of the students. So my kids just—like at first it was okay, but then as time went on they were just like, “Ech, I do not want to watch the teacher. I do not want to listen to the teacher. I just want to do the work.” *Parent* On our end the struggle was mostly getting like being motivated to actually get on and participate because it was so easy to kind of just slink back and not be a part of the learning. And because kind of even like in this small group if you have people talking over each other or whatever the case is then with kids it’s a lot harder for them to actually pay attention when they are being either talked over or they are getting muted or they have something to say but nobody is actually—they cannot actually hear it. *Parent* Well, I feel it was kind of confusing and then, like, the teachers went fast … like, all the time, when like, it was online, all the teachers would, like, do the lessons and stuff and it would be, like, kind of fast, and maybe it was because the schedule. *Student* It was really difficult, I’d say, because you did not really get much of the materials because the teachers could not really explain it. *Student* A con for online would be not really understanding the assignment. Yeah, mainly not understanding the assignment, which, in class, you mostly can ask questions during—well, you could ask questions during online, too, but it’s more, I would say, difficult to do that online. *Student* We tried our best to provide (educators) a lot of professional development and training and devices and the technology that was needed, but that did not have as much of an impact as we hoped. *School Employee* For teachers … who have used technology in their instructional practices, and have been taught how to use it, and grown up in a world where technology is part of their everyday lives, they adapted very quickly. Many of the things that they had been asked to do as students, maybe in colleges and universities, they had already done. And it was not that big of a deal. Some of them had already been involved in distance learning-type courses. And so, for them … the change was not as great. But if you get into teachers that are 15, 20, 25 years into the profession, they have never had any of that training, they do not do that, they do not feel comfortable at it, and they struggled with it—very much so. *School Employee* As far as actual learning that took place, we struggled a little bit as a district to find our footing in navigating having students online and in-person at the same time …. Everything we did we tried to do, obviously, in the best interest of the kids, but I think that that probably had to be really difficult …. Knowing when you could have time with your teacher to ask those questions that you needed. Getting help typically we would have tutoring so that if students did not quite get it during class, they would have afterschool and we were not able to offer that. *School Employee*
Subtheme 1c: student motivation and engagement was negatively impacted	They (school counselors) knew what my son was going through and me as a single parent what we were going through. So, they were willing to say, “Hey son. If I need to come out to your house and knock on the door to make sure that you are logging in because I know your mom is at work I will do that.” And I was grateful because I trusted him as a male counselor figure to come out and speak to my son to let him know that the decisions he was making were going to be more detrimental to him in the long run if he just kept letting it pile up. Because I could tell him everything and get blue in the face, but I wasn’t getting anything done. And I could not log in for him to do his work because that’s not teaching him anything. *Parent* In my case, you know them, I have two daughters and the worst problem was that each of them would go study to a separate room. I have internet, but each of them in their room. The oldest one graduated this year, but she barely graduated because according to her, she was studying, but she was sleeping and we think they are studying. We do not want to open their door so we do not interrupt them, but on several occasions, I was able to open the door and she was in deep sleep. She was snoring and her computer was on …. If I told her something: “I know what I’m doing, Mom. I know what I’m doing.” *Parent* There were not many pros to online learning. None. There were just, it was just easier to get distracted. Very easy to get distracted, easy to slack off ‘cause no one is watching you. I mean it’s not like they are going to do anything. And it generally just encourages laziness. *Student* (Reasons for logging on to class late or not at all) lack of motivation, because sometimes you just get lazy and do not want to do it. *Student* So I know that I had a few families or parents when I would reach out to them and say, “Hey, so-and-so has not come to school. We’re just concerned. We do not want for them to fall behind. We would hate for next year to come and them be really behind, and it’s really important for them to come.” “Well, I’m at work. I cannot be at home and making sure that they are logging on. I cannot be at both places at one time.” You know, and there was a lot of that. A lot. “I call them and I asked them to log on, they have said they have logged on, and then you guys are calling me and telling me they are not online.” *School Employee* My oldest is in high school, and the pandemic has been extremely, extremely difficult for him. Because he does not do well with self-guided online learning, and he really, really struggled, stopped making a lot of effort in being in school or even trying to attend his classes. He is gonna graduate, so that is huge, but it took a tremendous emotional toll on his confidence, on his self-esteem. *School Employee*

Theme 2: schools implemented numerous COVID-19 mitigation strategies — adding COVID-19 testing may send them “over the edge”	
Subtheme 2a: mitigation strategies have been implemented, there is widespread support among parents and students, and school employees take pride in their work to prepare schools for safe reopening	That they maintain distancing more than anything. They should always recommend that they wash their hands. If it’s possible, they should always cover their mouth, keep the mask on. I think that’s the main thing. *Parent* If they do not have a mask or if the string broke, they’ll give him one. Uh-huh, because he’s come home with a different mask and I tell him, “You were not wearing this one.” He said, “The teacher gave it to me.” And she gives him bottles of sanitizer. *Parent* So, in the beginning (following preventive measures at school) was a bit annoying because you had to do it all the time. But you’d get used to it once you went to school. *Student* There’s not much to complain about (regarding preventative measures at school). I mean it keeps everybody safe. *Student* The idea of social distancing, I think, is a good preventive measure, okay, and I do agree with the science that that and masking are things that we can do to protect ourselves. If not vaccinated, obviously. But the social distancing of six feet is the biggest challenge for us. It does not allow us to educate students in a way that we think is in the best interest of their education. So, we know that, you know, obviously, the three-foot distance was adjusted, and that is helpful and that allowed us to bring more students back in. But the six feet of social distancing during mealtimes is absolutely impossible for us, to be able to run a school and meet the needs of our students. Because we cannot use our facilities in the way that they were intended to be used. And kids, especially in the elementary, then, are asked to eat lunch in classrooms. Well, they do not normally eat lunch in classrooms, so we run into all kinds of staffing-related issues with regards to that. *School Employee* I’m a little concerned to be honest with you because…there is no way to keep them six feet and if we go to the three feet, but I still have to contact trace within six feet. That makes no sense to me whatsoever, to be honest with you because now I’m quarantining twice as many students as I did before. Sometimes I do not even have to quarantine any because they have stayed away the six feet, but now I would have to quarantine twice as many kids or more, which is more phone calls and more conversations with parents who are already ticked off ‘cause their kids are missing so much school because of the COVID thing. So, I’m really concerned about that, if I’m honest. *School Employee* And we have been blessed with what I think is a very supportive health district, and they were very happy to come down and walk through our buildings and look at what we had put in place, and then provide us guidance on how adjust to make it even better, or to praise us in what we were doing. And so, we felt better, as a district, about how we were addressing those health and safety measures. *School Employee*
Subtheme 2b: mitigation measures will prevent the spread of COVID-19 at schools; therefore, regular testing is not needed	If they have (children) overcome all the difficulty of the pandemic during all this time without a test, I mean, I do not think it will be something essential to—just participating in a test, well, I would definitely leave it up to them and I think none of them would accept, because as number 4 said, I also think it’s very uncomfortable for them to get it every week and what’s the point of them getting it when I think, they have already overcome the difficult parts of the pandemic without getting it and they have already gone to school for a fair amount of weeks when the rate was still high and nothing happened, so now, they even consider it unnecessary. *Parent* Personally, I do not feel that it’s necessary. I think that we had an attestation system we had in place since students came back in person and it worked very well. You know, we had—as a parent, I felt that that was adequate. I want to be able to trust our families and I want the school district to be able to trust me as a parent. As an admin, I also feel that it’s not necessary just because I know the safety precautions that we do have in place. I know the amount of time and energy it took to bring in those attestations and do the temperature checks. The limited number of cases that we did have, I do not think that something like that would be necessary. *School Employee* I guess I’m the under—I’m not an over worrier I guess. I do not know if that makes sense. We are maintaining the distancing, maintaining the cleanliness, always—we are always doing that. I am not that concerned with kids catching COVID. If they do, they do. There are cases of kids catching COVID, but I feel like if all of those precautions are in place then that mitigates the risk of anybody else catching COVID within our school system. And I do not know of any instance of anybody catching COVID within our own school. *School Employee*
Subtheme 2c: schools lack resources to implement a COVID-19 testing program	There’s no way right now it’s a possibility with our current staffing. I cannot do it. I do not know who would do it. The unions would not allow it ‘cause they refused. All the other unions, said, they just like, “No, we are not doing any of that.” And they put it in their union contracts. So it would fall on me and there’s just me. I cannot. So I am totally against it, to be honest with you, just from a staffing point of view. *School Employee* Now, if it’s part of a school program, my greatest fear is not having the staffing to be able to support it. Our school nurses have worked harder than probably any other group that we have in our school district. We are lucky that we do have a school nurse at each of our eight schools, so we do not have a shortage of nurses, but I know they were overwhelmed. Not only by the responsibility that they had to take, but just the regular work. And so, if there’s not appropriate staffing to support a testing program, then I’m gonna have a mutiny from our nurses. (Laughs) they are not—they are gonna be challenged to do that. *School Employee* I think it’s a good idea (testing), but I’m unsure of who would do it. I know that for me I have 900 kids that … so to add that on top of medication administration, my diabetic. If they brought, would bring in somebody from the outside to do that, that would be great, but as an added assigned duty for a nurse in the school that would be challenging. *School Employee*

Theme 3: familiarity with COVID-19 testing procedures influences support for testing	
Subtheme 3a: perceived benefits of school-based COVID-19 testing	I would rest easier knowing—every week—that they aren’t at risk—and they could come home. It’s better knowing that they would come home well … without getting infected. *Parent* I think that as long as it’s for their safety and the safety of those at home—because I do not know how many will go to school infected and from how many more they could get infected. I would rather have them get it every Friday and by Monday, we know that they are not infected and for my safety, because I’m diabetic, I would prefer for her to get it every week. *Parent* I would like it because I would be sure that he does not have it. Now they say it does not hurt as much as when they put the cotton bud all the way up. They say that it’s different now. I would like that so I can be sure that my children are healthy. *Parent* (Student is willing to participate in testing because) well, by knowing that I’m safe it also helps people around me stay safe of COVID. And my family members. *Student* So, to benefit—because we take our temperatures, every day, every day before we walk in the building. To do that, I would—I think it would not hurt for safety reasons and to keep the virus in control and intact with the sanitizing and cleaning and the consistency, this would not hurt to do that. Now, the only thing about it is, I do not know how parents would feel. But as a teacher, I feel that if we had to have that nose swab, you know, weekly, we would catch it earlier and early so that we do not have this blown-out positive effects, I mean, that it would be affecting more students than we want to in the building. *School Employee* If testing can be a value-add and help families and our students become healthier, then we are for it. Because the healthier they are, the better that they are gonna be able to perform and to be able to learn. We know that if kids are not healthy, then that is gonna become their number one concern, and their learning is going to struggle because of their physical or social-emotional health. So, anything that we can do to support a healthier student, a healthier family, a healthier school district workforce, we are for. *School Employee*
Subtheme 3b: unfamiliarity with testing negatively influences support for COVID-19 testing at school	I do not want them to get tested at school. It hurt. My son had a temperature and they made him get a test, even though I knew he never had it. He never had—he never got—he wasn’t around anyone and they forced him to get the test and it really hurt him and I’m glad I was there with him when he had to take the test. I mean like some people say it’s nothing, it do not hurt, but I know it did, it hurt him. I do not want them being tested for COVID at school when I know they are not getting it anywhere from anyone. *Parent* For instance, I’ve heard that they put that thing all the way up there—at least with the testing that they used to do. I did not know that it was different now, but it did worry me that they would put the cotton bud all the way up the nose. That was really uncomfortable. *Parent* I think weekly is too much, because even though it’s not painful, it is like very bothersome. My kids have all gotten the test and I’ve taken them and I think it’s kind of bothersome. I think they would get upset too and they would not want to go to school. *Parent* My family got COVID-tested but it wasn’t the nose one. It was the swab, a Q-tip around your mouth all the way to the back of your throat. Yeah, it was like scary at first and it did hurt because he went all the way down my throat. *Student* I think the simplicity of the test (would encourage people to get tested). Uh, earlier you mentioned, you know, the-the initial, um, deeper, uh, process and, uh, most current now, the tickling of the nose. I have experienced both of them, and I know the-the first process was very, you know, painful to the point that I even left that, uh, time saying, my god, you know, they took out my brain, you know? To then, uh, recently and I did participate almost, uh, every 2 weeks, uh, just for my safety to go and do the most current one. So, I think simplicity, um, is one. And-and I think then, uh, identifying a-a method where we do not have to go so deep. Uh, I think that really, um, um, encouraged individuals. *School Employee* Honestly, it’s kind of hard to give a straight answer for that, because I’ve went through it a couple of times which was the long too like you said all the way in the back of your brain and stuff, it was really uncomfortable. *School Employee*
Subtheme 3c: parents need to be informed about COVID-19 testing procedures	I would definitely be concerned if they would be getting those swabs, actually their nose tested weekly. Especially since with my son his nose is very sensitive to the point where he gets bloody nose if he even touches it. So that’s something that I would be very concerned at least with my son where he would get bloody noses. Not only that but like the side effects of it. Like the previous person just said what about their skin. Would it get irritated? *Parent* I have a few concerns with that. I would like to know who is offering the testing? Is it the teacher? Is it the school nurse? Do they have medical qualifications to be providing the test? Are they doing the test accurately every time to make sure that it’s not missing something that could be there or not there like we said with the negatives or positives? To me it would be kind of really hard to get all of those students in such a short amount of time when they are supposed to be entering the building and leaving the building so that way we make sure there’s nothing missed. That’s my concern. *Parent* Oh, boy, I think we’d have to be really doing a lot of communication. We have to have data to support that (regular COVID-19 testing). You do have to have the data …. (having) Zoom meetings, I think it’s important so the parents can ask questions. Address these questions or concerns to the administration, to the teachers, educators, and ask these questions. And then we could respond to help them feel better that it is better for that—to do that than to wait for a whole month and it’s already blown out, see? And we’d probably need to, you need to have the medical field involved with this. You need to get doctors and nurses involved with questions, because they may have questions that we cannot, as teachers could answer. You know, the side effects of what may be a nose swab or, you know, they might think it’s gonna harm their child. *School Employee* I do not think I would want to participate in that (school-based COVID-19 testing). I think that that’s kind of an invasion of the kids’ rights and you would have to have parent permission and everything. But that would be—we find a lot of parents that they are not taking their kids to be tested because it was so invasive at the beginning. I do not know that they realize what it has evolved to. So maybe that’s a big part of communicating that to families what that would look like. But I, at this point I think that I would get a lot of push back and parents would not be on board to do that. *School Employee*

Theme 4: there are inadequate resources to support families after a positive COVID-19 test	
Subtheme 4a: no financial support for parents who need to take time off work	Our Hispanic, uh, families are primarily, uh, labor-working families. And that has not stopped, uh, them from getting out, having to go to work, having to purchase their food, having to, you know, do, uh, uh, things, um. But yeah, the assistance for our, our migrant- and farm-laboring families, um, have not really seen that support. Uh, but their demand has, has been there, yeah, yeah, 100 percent. Do not stop working. You still need to go to work. *School Employee* And then the worst thing too is … the child’s quarantine starts day ten of the parent’s quarantine, so the child is home for almost a month ‘cause they have been home the whole ten days of their parent’s illness and now they need to quarantine for ten days. And so that part, at least in our department, has been very difficult. And then parents … they finally get their jobs back (after completing quarantine), they finally are going back to work and all of a sudden, “Oh sorry, now you have to stay home with your kid for ten days.” *School Employee*
Subtheme 4b: quarantine and isolation is difficult for families to maintain at home	No. I do not think so because—like I said, I only have the one bathroom and—year—we depend on the one bathroom. And even if there were two, you have to walk through the house to go to the bathroom. It would be really hard, it would be—yeah, really hard. *Parent* We do not live in like—we do not have a separate garage. We do not have a separate—he has a separate room but you have to pass his room or he would have to share a bathroom because we only have one restroom. So that’s how I know I could not just fully quarantine and isolate him. *Parent* But if we talk about, um, overall population that we serve, um, and the families that I, uh, work with, um, essential agricultural workers, um, we have many of them that are doubled up in their—in, uh, one home. It’s, um—you know, uh, you know, we could definitely see where, uh, two families to a home. Maybe sometimes even three. Um, but if they were to—if we have a kiddo getting, um, uh, COVID and we send them home to-to be isolated, again, we—they have younger siblings, older siblings in-a home where it’s a two-bedroom, three-bedroom and, you know, they sleep together many of them. So again, um, they are truly not isolated *per se*. So that’s where I think we have seen, uh, some households where, uh, the whole family-gets, uh, um, you know, the virus. Because again, there’s very limited isolation. *School Employee* Because of the agricultural setting that we are in, it’s spread through those workers quickly and because there’s multigenerational households, so one person would go to work, get it, bring it home, give it to grandma and grandpa, the kids. They could not very, they could not separate in the house and quarantine very easily, because a lot of times you know there was 12 people in a two-bedroom home, so it spread quite rapidly in our area. *School Employee*
Subtheme 4c: getting food when parents need to quarantine or isolate	Even when I got the COVID test, the clinic calls to help you. They gave me a lot of food. There’s a—they got together with Fiesta Foods because every person who got a positive test result, they would give you a basket but not just any basket, a cart full of all types of food, everything you needed: rice, beans, meat, chicken, soup, Gatorades. I was even asked if I needed other things, yes, because they give you bleach, they give you supplies to wash your clothes and they even told me, “If you cannot pay your rent, we can pay your rent, your electric bill.” *Parent* As a mom, I have to go out to buy things; I mean, groceries, milk, and everything they have to drink, so I had to go out (when I had COVID), because they would call me from the clinic and they would ask me, “But, what store did you go to?” So, I would go to Walmart, I would have to go out, or are you going to send someone to leave my groceries here at home? Who’s going to do it? I have to go out. My daughter did stay in her room and she did not go out, but me, I felt frustrated, because I had to go out to buy my things for my children’s food, because even if I do not want to eat, I have to feed them. *Parent* It can be challenging, but I have … I had one family that the whole family got it, mom was unable to leave, because they were all quarantining. So, you know we took them some food and left it on the, on the doorstep and offered you know any assistance that we could, could give them. *School Employee* No, not quarantine, not based on what the expectation is of staying home for that duration of time. No, I do not think so. I think families still have to move to get their groceries, to get a lot of their services. Our community is not to the point where we can order food right from Walmart and have it delivered. We do not have a Walmart close to us. I know that some locations in the state and some families do have access to resources like that. In our community, I think people still have to be mobile in order to continue with the function of their family, so to maintain quarantine would be difficult. I think some families try their best, but they cannot. *School Employee*

### 3.1. Theme 1: there is a shared desire for students to return to in-person learning

All three participant groups agreed schools need to reopen as students experienced challenges with remote learning. During remote school hours, students needed to prioritize household chores, care for younger siblings while parents were at work, and assist siblings in their remote learning. A student shared that the biggest challenge to remote learning was “probably having to take care of my brother while learning.” Students, especially those from large households, did not have a quiet space free of distractions.

All three participant groups reported that remote teaching was of lower quality than in-person teaching as teachers were unaccustomed to using technology in their instructional practices. School districts provided training on remote teaching but reported that their efforts fell short. Numerous students said remote instruction was fast-paced, and they struggled to keep up and ask questions. Some parents reported that their children asked them for help, but parents with children in high school found the curriculum challenging and were unable to help.

Challenges with remote learning affected students’ motivation and engagement. Students logged on to class late or not at all and often turned in assignments late. While teachers and parents found this problematic, they also found it difficult to hold students accountable, given what they were going through. A school staff member said that students would say, “How do they want us to concentrate, and we have to be taking care of the other kids?” Working parents reported feeling guilty. One said, “I feel I failed my child…I worked two jobs during the pandemic … I wasn’t at home as much to be that parent that needed to make sure that they were logging in on time, making sure that they were completing their assignments.”

### 3.2. Theme 2: schools implemented numerous COVID-19 mitigation strategies—adding COVID-19 testing may send them “over the edge”

School staff described implementing numerous COVID-19 mitigation strategies, including social distancing, masking, disinfecting, daily screening for COVID-19 symptoms, and contact tracing. While parents and students were supportive of the mitigation strategies, there were mixed feelings about adding school-based COVID-19 testing as a mitigation strategy. Many school staff and parents believed that the existing mitigation strategies were effective at preventing outbreaks and therefore adding testing was unnecessary. Some school staff and parents believed testing should be optional and only administered to students with parent permission. On the other hand, students were supportive of COVID-19 testing because they viewed it as a step toward returning to normal life.

Many school staff shared that it would not be feasible to take on the responsibility of COVID-19 testing as they were already overworked from responding to the pandemic. A school nurse explained, “Our health services team is stretched so thin right now … even asking us to do one more thing I think is going to send some of the nurses over the edge.” In addition, a school-based COVID-19 testing program would further shift schools’ responsibilities from education to health. A school administrator stated, “We are not staffed or resourced appropriately for that. And that’s probably one of my greatest fears of schools becoming responsible for health as well as education.”

### 3.3. Theme 3: familiarity with COVID-19 testing procedures influences support for testing

All three participant groups were used to COVID-19 mitigation strategies such as masking and social distancing, but not testing. Many parents, school employees, and students reported that they had never been tested for COVID-19. Others had been tested via oropharyngeal or nasopharyngeal swabs or heard negative experiences from others. All three participant groups described COVID-19 tests as invasive, painful, uncomfortable, and bothersome, and they were less supportive of regular, school-based testing. A school nurse believed the community would be more supportive of regular testing if samples were collected with nasal swabs. The nurse shared that 80%–85% of parents declined COVID-19 testing for their children, which at the time used nasopharyngeal swabs, because they did not want to subject their children to a painful procedure.

In addition, parents were concerned about repeated swabbing. For example, one parent said, “My only concern is if they are going to continually take a sample from the same area … that might cause irritation or a rash or like a pimple.” School employees noted that if schools were to implement a COVID-19 testing program, they must first address parents’ concerns about testing to gain their support and participation. School employees and parents wanted health professionals (not educators) to provide information about the testing procedure, including qualifications of test administrators, sample collection, and interpretation of results given the likelihood of a false positive/negative.

### 3.4. Theme 4: there are inadequate resources to support families after a positive COVID-19 test

School staff and parents explained that many parents do not have remote jobs or paid time off, making it difficult to stay home if their child has COVID-19. One staff member stated, “You know if you do not go to work … sadly you do not get paid. So, I can still see that as being an issue in our community.”

In addition, adhering to the recommendation to isolate after a positive COVID-19 test was deemed nearly impossible for many families. A school staff member explained, “I can tell you that 60% of (families) here in our community are doubling up, tripling up (in a single home). It would make it very hard for somebody to isolate in their home.” When students test positive at school, they isolate in the school’s holding room until a parent/guardian picks them up. Once students go home, however, they cannot easily separate themselves from family members to maintain isolation. School staff further shared that transmission among entire households was common.

Some parents and school employees felt it would be difficult for parents to maintain isolation at home because of the need to obtain food and other necessities. This was highlighted as a potential barrier to participating in COVID-19 testing and following isolation recommendations after a positive test. While some participants mentioned that people could get food delivered to their home (e.g., from grocery stores, food banks, family members, and school employees), others reported minimal access to food delivery services, especially for migrant and farmworker families and those living in rural areas of Yakima County.

## 4. Discussion

In our qualitative analysis to understand parent, student, and school employee perspectives on school-based COVID-19 testing, we identified four main themes. First, there was a desire for schools to reopen because remote learning was challenging for teachers, parents, and students. Second, a perceived lack of resources led school employees to oppose the idea of adding school-based COVID-19 testing to the existing COVID-19 mitigation strategies. Schools prepared for safe reopening but were strained by their new responsibility for children’s health. School employees and parents believed existing mitigation strategies were sufficiently effective at preventing COVID-19 outbreaks. Third, parents were reluctant to let their children undergo regular testing as many parents had never been tested and believed the procedure would be painful. Fourth, following isolation recommendations after a positive COVID-19 test was seen as untenable since many parents did not have paid time off, homes were not conducive to isolation, and food delivery was inaccessible in rural communities.

Previous studies have examined parent, student, and school employee perspectives on schools reopening and implementing COVID-19 mitigation measures (22–27). While our study found a widespread desire to return to in-person learning, previous studies report mixed findings. The studies found that some parents, students, and school employees were ready for school to reopen, while others were concerned about the risk of COVID-19 transmission in schools and preferred to continue remote learning (22–26). Similar to our study, the previous studies also report mixed community perspectives on using COVID-19 testing to support safe school reopening (24, 25, 27). The studies found that teachers and other school staff were receptive of school-based COVID-19 testing (24, 25), while school administrators questioned the need for testing and were concerned about the cost and logistics of incorporating testing into their reopening plans (25). Furthermore, a qualitative study conducted in an urban region of Southern California, where school districts have a large population of Hispanic/Latino students, found that students were supportive of school-based COVID-19 testing but were concerned about the physical discomfort of testing (25). These students’ parents also worried about the children experiencing physical discomfort while undergoing COVID-19 testing (25). Generally, however, the previous studies report that parents were supportive of school-based COVID-19 testing (22, 24, 25). The parents in our study were hesitant about COVID-19 testing in schools because of the perceived physical discomfort their children would experience. While these findings may be interpreted as parents’ distrust of the medical system, they may also be biased by the testing technology. At the time of our data collection, COVID-19 testing commonly used nasopharyngeal swabs, which can be perceived as more unpleasant relative to other less invasive sample collection methods (28).

Few studies have examined community perspectives on COVID-19 mitigation efforts in schools (22–27), and we could not identify studies focused on Hispanics/Latinos in rural regions of the U.S. For COVID-19 mitigation efforts to succeed, communities must support these efforts and have access to resources to adopt the recommended behaviors (6, 23). This study with mostly Hispanic/Latino participants in Yakima County extends our understanding of Hispanic/Latino communities’ acceptance of school-based COVID-19 testing (25). Our study found that school employees were apprehensive about the responsibility of children’s health being increasingly shifted to the purview of schools during the COVID-19 pandemic, especially since they had inadequate resources and were not medical experts. COVID-19 testing was not widespread in Yakima County at the time of data collection, and the lack of familiarity with testing procedures may have negatively influenced support for testing in schools. Public health practitioners should consider building partnerships with schools to understand their concerns and provide adequate resources that equip schools to address these concerns.

This study has limitations worth noting. The data was collected from June to July 2021, and perceptions on COVID-19 testing in schools may have since changed given the rapid and ever-evolving nature of the COVID-19 pandemic. Nonetheless, the study findings are relevant and important for understanding the acceptance of public health measures and the resources needed to adopt them in a population that is under-represented in research and often disproportionately burdened by disease. Future research is needed to examine how community perceptions of school-based COVID-19 testing have evolved since schools initially reopened for in-person learning.

Our findings suggest that school employees would support school-based COVID-19 testing if adequate resources and healthcare professionals are available to implement the testing infrastructure. In addition, we found a preference for healthcare professionals, not educators, to provide evidence-based COVID-19 testing information to the community. Finally, our findings show that many families do not have the physical space for a child to isolate, parents must decide between quarantining/isolating or losing income because they do not have paid time off or the ability to work remotely, and parents may not be able to follow isolation recommendations because of limited alternatives to in-person shopping for food and other essentials in rural regions where food delivery is inaccessible. COVID-19 testing policies must consider and help mitigate the downstream effects on households when a child and their family members test positive. Implementing testing programs without adequate planning and resources for those who test positive could unintentionally create a net decrease in well-being within some communities. It is critical that COVID-19 testing programs provide families with the resources needed to isolate after a positive test. Implementing properly resourced school-based COVID-19 testing programs could be an opportunity to form school–community linkages and pool community resources in rural regions so that families can follow isolation guidelines and still have access to basic needs like income and food (29).

## Data availability statement

The raw data supporting the conclusions of this article will be made available by the authors, without undue reservation.

## Ethics statement

The University of Washington Institutional Review Board approved the study. Parental consent and child assent for student participants were obtained alongside verbal or electronic informed consent to participate in this study.

## Author contributions

MR led the analysis of the coded excerpts, drafted the initial manuscript, and revised the manuscript based on co-author feedback. SB conducted school employee interviews, supervised data collection from students and parents, and supervised and had oversight of the data analysis team. GI contributed to the methodology and data curation. PS consulted on study design, methodology, and resources and had supervision/oversight of study staff. MD assisted with data curation and formal analysis. HC contributed to data curation and formal analysis. LH managed administrative aspects of the study, liaised with the national coordinating center, mentored student workers, and supported the investigative team. LG and SL contributed to the methodology, investigation, funding acquisition, data curation, and formal analysis. MG consulted on study design. PD contributed to the study investigation. LK contributed to the conceptualization of the study, methodology, investigation, supervision, funding acquisition, data curation, and formal analysis. All authors contributed to the article and approved the submitted version.

## Funding

This publication was supported by the National Institutes of Health under Award Number 1OT2HD107544-01. This research was also supported by the Office of Community Outreach & Engagement of the Fred Hutch/University of Washington/Seattle Children’s Cancer Consortium (P30 CA015704). The content is solely the responsibility of the authors and does not necessarily represent the official views of the National Institutes of Health.

## Conflict of interest

The authors declare that the research was conducted in the absence of any commercial or financial relationships that could be construed as a potential conflict of interest.

## Publisher’s note

All claims expressed in this article are solely those of the authors and do not necessarily represent those of their affiliated organizations, or those of the publisher, the editors and the reviewers. Any product that may be evaluated in this article, or claim that may be made by its manufacturer, is not guaranteed or endorsed by the publisher.
